# Some Penal Cases

**Published:** 1917-12-15

**Authors:** 


### SOME PENAL CASES.

The November session of the General Medical Council rarely arrives without a record of one or more instances in which the Council has been obliged to exercise its penal functions and to erase the name of the offending practitioner from the Official Register. The duty is a painful one, but the public interest demands its discharge, and, indeed, this interest is the only justification for such action. The title "registered" medical practitioner is an announcement of character as well as of professional qualification, and the Legislature has imposed on the Council the duty of keeping the list "clean" in this respect. Necessarily the standard adopted is a more or less conventional one, and is adjusted not so much to guarantee personal excellence as to catch the open and notorious offender. "Infamous conduct in a professional respect" is the technical verdict delivered against the sinning practitioner, and the phrase has a truly sinister sound. It may be open to criticism as suggesting an offence against arbitrary and professional rules, but its expression and the penalty it involves can only be defended when they are in harmony with the general welfare. In essence, the offence is conduct which shows the practitioner concerned to be a person unworthy of public confidence, and therefore unsuitable for the practice of a profession in the practice of which this confidence is in a special degree invited.

At the recent meeting of the Council several cases came into this category, and the findings, granted the position just stated, will hardly be challenged. In one instance a practitioner was found guilty of continuing over a period of six months to certify a man as unfit for military service, although, except during the first fortnight of this period, he had not examined the man or even seen him. A second case involved a charge of supplying to a woman patient, and at exorbitant prices, large quantities of a "narcotic toxicant drug named heroin." Apart from the moral aspects of the accusation, there was here an offence against the Defence of the Realm Regulations, as the drug, at least in part, was alleged to be for the use of "members of His Majesty's Forces." There can be no surprise that the verdict of guilty involved the removal of the offender's name from the Medical Register.

Two other cases which produced this penalty belonged to the very serious group in which the practitioner is found to have committed adultery with a married woman to whom he had acted as medical adviser. Naturally, the latter offence is regarded as a very aggravated one. No one with a knowledge of the world will imagine that in such instances the temptation is invariably inaugurated by the practitioner or that he is always guilty of having led innocence astray. But in this particular respect the medical profession must reach a standard of conduct higher than that demanded without qualification from ordinary mortals. Among the latter Joseph is quoted as reaching an unusual level of resistance to temptation, and his achievement is consequently put upon record. But what may be unusual elsewhere must be invariable in the medical profession. Its members are trusted in very delicate situations, and the measure of this trust is the measure of their obligation. The obligation is recognised without stint, and it is freely and honourably met. When, as does occasionally happen, this trust is betrayed, there is no circumstance which can be pleaded in mitigation of the professional offence. The moral blame may vary in different cases, but the professional standard in such matters is that the practitioner shall neither institute temptation nor yield to it. If the offence is committed, whatever be its genesis, there is only one possible penalty—namely, expulsion from the lists of a responsible and honourable profession.

